# Draft Genome Sequence of a Metronidazole-Susceptible *Atopobium vaginae* Isolate

**DOI:** 10.1128/genomeA.00991-15

**Published:** 2015-09-03

**Authors:** Jessica A. Schuyler, Eli Mordechai, Martin E. Adelson, Scott E. Gygax, David W. Hilbert

**Affiliations:** Femeris Women’s Health Research Center, Medical Diagnostic Laboratories, A Member of Genesis Biotechnology Group, Hamilton, New Jersey, USA

## Abstract

We report the draft genome sequence of a vaginal isolate of *Atopobium vaginae vaginae* (strain 44061), an organism linked to bacterial vaginosis (BV), the most common gynecological infection in the United States. This species is often highly resistant to metronidazole, which is a front-line therapy for BV. Strain 44061 is a metronidazole-susceptible isolate (MIC, 16 µg/ml), and its genome sequence will be useful for comparative studies to elucidate the molecular basis of metronidazole resistance in this species.

## GENOME ANNOUNCEMENT

*Atopobium vaginae* is strongly associated with bacterial vaginosis (BV) (1), the most common cause of vaginal discharge in the United States (2). The antibiotic metronidazole is recommended by the Centers for Disease Control and Prevention as a front-line therapy for BV (3), but treatment failure is common (4), generating interest in evaluating metronidazole susceptibility of BV-associated organisms. *A. vaginae* is often highly resistant to metronidazole (5), including the sequenced strain DSM 15829 (GenBank accession number NZ_ACGK02000000), which has a MIC of >256 µg/ml when 32 µg/ml is considered a standard breakpoint for metronidazole resistance (6). For comparative studies, we sequenced the genome of 44061, an isolate we found to be susceptible to metronidazole (MIC, 16 µg/ml).

Isolate 44061 was obtained from the Culture Collection of the University of Goteborg. It was originally isolated from the fornix of the vagina from a woman with bacterial vaginosis. We determined the metronidaozle MIC using a metronidazole Etest strip (bioMérieux). Genomic DNA was isolated using a Gentra PureGene kit (Qiagen). Genomic DNA was sequenced using the 454-GS Junior System (Roche) following the manufacturer’s instructions, and genome assembly was performed using the GS De Novo Assembler (version 3.0). The NCBI Prokaryotic Genome Annotation Pipeline (http://www.ncbi.nlm.nih.gov/genome/annotation_prok/) was used for genome annotation.

We generated 91,698 reads totaling 41,189,768 nucleotides. These reads were assembled into a draft genome of 1,483,295 nucleotides with 26-fold coverage. The genome encodes 1,267 genes, 3 rRNA operons, 45 tRNA genes, 109 pseudogenes, 1 noncoding RNA (ncRNA), and 1 clustered regularly interspaced short palindromic repeat (CRISPR) array. We compared the genome sequence to that of DSM 15829 using GS Reference Mapper (version 3.0) and found 25,419 high confidence differences (i.e., nucleotide polymorphisms). In addition, we found structural variations including 10 deletions, 15 duplications, 16 insertions, 44 substitutions, one inversion, and one translocation. Further analysis of this genome in comparison to genome sequences of metronidazole-resistant *A. vaginae* isolates may help identify molecular determinants of metronidazole resistance in this species.

### Nucleotide sequence accession numbers.

The whole-genome shotgun project has been deposited in GenBank under the accession number LFWE00000000. The version described in this paper is version LFWE00000000.1.
